# Modification effect of multiple gestation on the relationship between maternal body mass index and miscarriage in frozen-thawed embryo transfer: a retrospective cohort study

**DOI:** 10.1186/s12958-026-01586-1

**Published:** 2026-07-04

**Authors:** Zhuoye Luo, Lili Wang, Zhiming Zhao, Guimin Hao, Rui Xing, Yinfeng Zhang, Shuo Huang, Aimin Yang

**Affiliations:** 1 Department of Reproductive Medicine, The Second Hospital of Hebei Medical University, No. 215 of Heping West Road, Xinhua District, Shijiazhuang, 050051 China; 2 Hebei Center for Quality Control and Management of Human Assisted Reproductive Technology, Shijiazhuang, China; 3 Hebei Key Laboratory of Infertility and Heredity, Shijiazhuang, China; 4 Department of Reproductive Medicine, Tianjin Central Hospital of Gynecology Obstetrics, Tianjin, China; 5 Department of Obstetrics and Gynecology, Center for Reproductive Medicine, Peking University Third Hospital, Beijing, China

**Keywords:** Frozen-thawed embryo transfer, Body mass index, Miscarriage, Twin pregnancy, Elective single embryo transfer

## Abstract

**Background:**

Elevated maternal body mass index (BMI) increases miscarriage risk in assisted reproductive technology, but it is unclear whether this risk differs between singleton and twin pregnancies. Twin gestations impose greater metabolic and hemodynamic demands, which may amplify the adverse effects of elevated BMI. This study aimed to determine whether pregnancy plurality modifies the association between maternal BMI and miscarriage risk following frozen-thawed embryo transfer (FET).

**Methods:**

In this retrospective cohort study, 13,911 FET cycles performed at a tertiary reproductive medicine center between January 2019 and June 2024 were included. Maternal BMI was categorized as underweight (< 18.5 kg/m²), normal weight (18.5–24.9 kg/m²), overweight (25.0–29.9 kg/m²), and obese (≥ 30.0 kg/m²). The primary outcome was total miscarriage (pregnancy loss before 28 weeks), with secondary outcomes including early miscarriage (< 12 weeks), late miscarriage (12–28 weeks), clinical pregnancy and live birth. Generalized estimating equation (GEE) models were used to estimate adjusted odds ratios (aORs) with 95% confidence intervals (CIs), accounting for repeated cycles from the same patient. Subgroup analyses stratified by pregnancy plurality (singleton vs. twin) and interaction tests were performed to evaluate whether plurality modifies the association between BMI and miscarriage.

**Results:**

Overall, early and late miscarriage rates were 11.71% and 4.59%. After adjustment, obesity (BMI ≥ 30 kg/m²) was significantly associated with increased risks of early miscarriage (adjusted odds ratio [aOR] 1.74, 95% CI 1.31–2.31), late miscarriage (aOR 2.35, 95% CI 1.55–3.57) and total miscarriage (aOR 2.04, 95% CI 1.58–2.62) compared with normal-weight women. Overweight women also exhibited elevated risks of late miscarriage (aOR 1.66, 95% CI 1.30–2.12) and total miscarriage (aOR 1.30, 95% CI 1.13–1.50). Subgroup analyses demonstrated that the association between elevated BMI and total miscarriage was significantly stronger in twin pregnancies than in singleton pregnancies (*P* for interaction = 0.018). Increasing BMI was not associated with clinical pregnancy or live birth.

**Conclusions:**

Elevated maternal BMI was associated with an increased risk of miscarriage after FET, and this association may be more pronounced in twin pregnancies. These findings highlight preconception weight optimization and support elective single embryo transfer in overweight and obese patients.

**Supplementary Information:**

The online version contains supplementary material available at https://doi.org/10.1186/s12958-026-01586-1.

## Background

Assisted reproductive technologies (ART) are increasingly used worldwide, and frozen-thawed embryo transfer (FET) has become a key strategy, offering improved endometrial receptivity and reduced ovarian hyperstimulation risk [1]. However, pregnancy loss remains a major obstacle to ART success.

Maternal body mass index (BMI) is a known determinant of reproductive outcomes. Obesity, highly prevalent among women of reproductive age, is associated with infertility, miscarriage, and perinatal complications [2]. In ART, elevated BMI correlates with lower implantation and live birth rates and higher miscarriage risk, likely due to metabolic dysfunction, insulin resistance, and impaired endometrial receptivity [3]. Pregnancy plurality also influences outcomes. Twin pregnancies are associated with higher risks of adverse pregnancy and neonatal outcomes as well as maternal morbidity compared with singleton pregnancies [4]. Although elective single embryo transfer (eSET) has reduced multiple gestation rates, double embryo transfer is still practiced in some settings.

Importantly, elevated BMI and twin gestation often coexist. Obese patients may undergo double embryo transfer to improve cumulative pregnancy rates, increasing the likelihood of twin pregnancies [5]. The greater physiological burden of a twin gestation includes heightened insulin resistance and cardiovascular strain, which can further amplify obesity-related metabolic dysfunction, potentially exerting synergistic adverse effects on placental development and pregnancy maintenance [6].

However, few studies have explored whether the effect of BMI on miscarriage varies by plurality. Understanding this interaction is clinically relevant for optimizing embryo transfer strategies. If elevated BMI confers greater risk in twin pregnancies, strategies such as eSET may be particularly beneficial for overweight or obese patients.

This study was designed to determine whether the effect of maternal BMI on miscarriage risk after FET differs between singleton and twin pregnancies.

## Materials and methods

### Study design and population

**Fig. 1:**
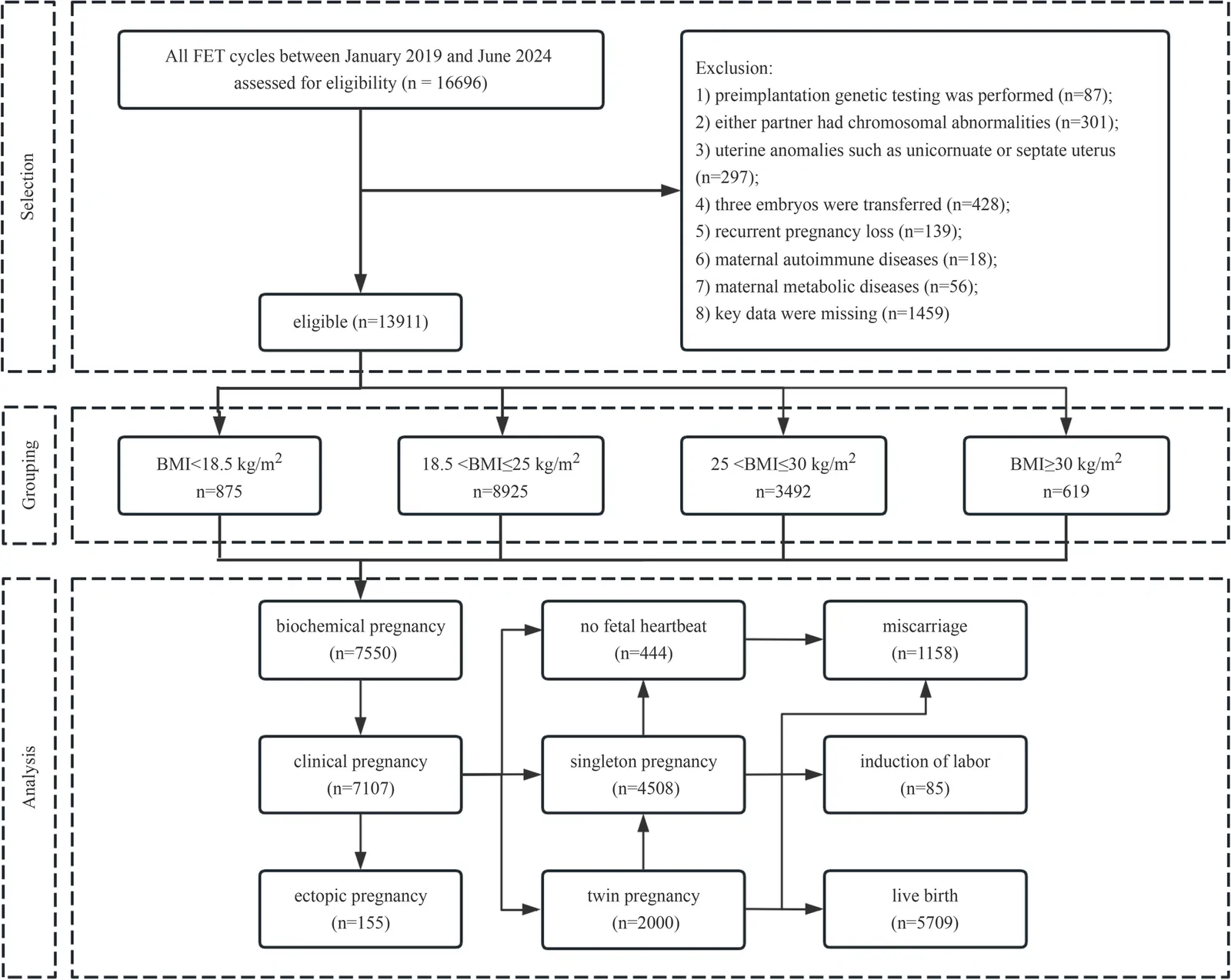
Flow chart of participant selection, grouping and pregnancy outcomes in frozen embryo transfer cycles

This retrospective cohort study included all FET cycles performed at the Second Hospital of Hebei Medical University between January 2019 and June 2024. Eligible cycles were identified from our ART database. Cycles were excluded if they met any of the following criteria: (1) Preimplantation genetic testing (PGT) was performed. (2) Either partner had chromosomal abnormalities. (3) Uterine structural abnormalities were present, including unicornuate or septate uterus. (4) Three embryos were transferred. (5) Recurrent pregnancy loss (defined as the spontaneous loss of two or more pregnancies which excluded confirmed molar or ectopic pregnancies) [7] (6) Maternal autoimmune diseases were present, including antiphospholipid syndrome [8], systemic lupus erythematosus, rheumatoid arthritis, etc. (7) Maternal metabolic diseases were present, including chronic hypertension, pregestational diabetes mellitus, etc. (8) Essential data were missing, including maternal BMI or pregnancy outcome information. After applying these criteria, 13,911 FET cycles were included in the final analysis. The selection of eligible cycles is illustrated in the flowchart (Fig. 1).

The study protocol was approved by the ethics committee of the Second hospital of Hebei Medical University (2026-R458), and the requirement for informed consent was waived due to the retrospective nature of the study.

### BMI assessment

Maternal height and weight were measured by trained nurses within one month prior to the initiation of the FET cycle. BMI was calculated as weight in kilograms divided by height in meters squared (kg/m²). Participants were categorized into four groups: underweight (< 18.5 kg/m²), normal weight (18.5–24.9 kg/m²), overweight (25.0–29.9 kg/m²), and obese (≥ 30.0 kg/m²) according to World Health Organization criteria [9]. For sensitivity analysis, BMI was additionally categorized using Asian-specific criteria: underweight (< 18.5 kg/m²), normal weight (18.5–22.9 kg/m²), overweight (23.0–27.4 kg/m²), and obesity (≥ 27.5 kg/m²) [10].

### Controlled ovarian stimulation and FET protocols

Controlled ovarian stimulation was performed using Gonadotropin-releasing hormone (GnRH) agonist (including luteal-phase and early-follicular-phase long protocols) or antagonist protocols. The specific protocols are detailed in previous studies [11]. Embryo morphology was assessed daily according to the Istanbul consensus (2011 version) [12]. Cleavage-stage embryos were cultured to day 3, and blastocysts were graded based on the Gardner criteria [13] and cultured to day 5 or 6. Good-quality cleavage-stage embryos were defined according to the updated Istanbul consensus (2025 version) [14]; good-quality blastocysts were defined as those with a Gardner grade of ≥3BB [15]. All embryos were cryopreserved using vitrification. For patients with height < 150 cm, weight < 40 kg, a scarred uterus, cervical insufficiency, or a history of twin miscarriage/preterm birth, single embryo transfer was recommended. For FET, endometrial preparation was conducted via natural cycles, hormone replacement therapy (HRT), or ovulation induction. Specific procedures for each preparation method, including monitoring strategies, medication administration, timing of embryo transfer, and luteal phase support, are elaborated in detail in previous studies [11].

### Outcome definitions

Pregnancy outcomes were defined according to standard clinical criteria: Biochemical pregnancy was defined as a serum β-hCG level greater than 5 IU/L measured 12–14 days after embryo transfer. Clinical pregnancy was defined as the presence of at least one gestational sac detected by ultrasound or villous tissue confirmed by histology test at approximately 4–5 weeks after embryo transfer. Early miscarriage was defined as pregnancy loss before 12 weeks of gestation, whereas late miscarriage was defined as pregnancy loss between 12 and 28 weeks of gestation. Total miscarriage included both early and late pregnancy loss. Live birth was defined as delivery of at least one live infant after 28 weeks of gestation. Pregnancy plurality (singleton or twin pregnancy) was determined according to the number of fetal heartbeats observed on ultrasound at approximately 4–5 weeks after embryo transfer. Cases with two gestational sacs but only one fetal heartbeat were classified as singleton pregnancies, whereas cases with two fetal heartbeats were classified as twin pregnancies.

### Statistical analysis

To handle missing data, multiple imputation was performed using the k-nearest neighbors (kNN) algorithm (k = 5), implemented via the VIM package in R. Missing proportions are shown in Supplementary Figure 1. Continuous and categorical variables were imputed using the mean and mode of the nearest neighbors, respectively. All predictor variables were included in the distance calculation without scaling. Continuous variables were expressed as mean ± standard deviation and compared using one-way analysis of variance or the Kruskal-Wallis test where appropriate. Categorical variables were presented as frequencies and percentages and compared using Pearson’s chi-square test or Fisher’s exact test. Because some women contributed more than one FET cycle during the study period, observations were not independent. To account for the correlation between repeated cycles from the same patient, generalized estimating equation (GEE) models with a logit link function, binomial distribution and an exchangeable correlation structure were used to estimate associations between BMI and reproductive outcomes. Both crude and adjusted odds ratios (ORs) with 95% confidence intervals (CIs) were calculated. The covariates were selected based on either their association with the miscarriage outcome of interest or their ability to alter the effect estimate by more than 10%. Multivariable models were adjusted for potential confounders including female age, infertility type, infertility duration, infertility cause, FET protocol, endometrial thickness, number of embryos transferred, embryo stage and embryo grade. Multicollinearity was assessed using the generalized variance inflation factor (GVIF) via the car package in R. All adjusted GVIF values were below 2, indicating no severe multicollinearity. To evaluate potential effect modification by pregnancy plurality, subgroup analyses were conducted for singleton and twin pregnancies. Interaction terms between BMI categories and pregnancy plurality were included in the GEE models, and the significance of interaction was assessed using the likelihood ratio test. To assess the robustness of the findings, sensitivity analyses were performed using Asian BMI criteria and excluding spontaneous twin reduction at 4–5 weeks after embryo transfer. All analyses were performed using R software version 4.4.0. Two-sided *P* values < 0.05 were considered statistically significant.

## Results

### Baseline characteristics

A total of 16,696 FET cycles were screened. After exclusion, 13,911 cycles were included for analysis and divided into four BMI subgroups. A total of 7,107 clinical pregnancies were observed, including 4,508 singleton and 2,000 twin pregnancies. There were 1,158 miscarriages and 5,709 live births in the overall cohort. The study flowchart and full details are presented in Fig. 1.

The mean maternal age was 30.68 ± 4.38 years, with a mean maternal BMI of 23.31 ± 3.55 kg/m². Primary infertility accounted for 53.15% of cycles, with a mean infertility duration of 4.34 ± 2.97 years. Tubal factor (51.32%) was the most common infertility diagnosis, followed by male factor (14.38%) and polycystic ovary syndrome (9.03%). Hormone replacement therapy was used for endometrial preparation in 74.65% of cycles. The mean endometrial thickness was 9.63 ± 1.65 mm. Two embryos were conducted in 79.35% of cycles, and 78.70% of transferred embryos were at the cleavage stage. A total of 3,741 cycles (26.89%) had no good-quality embryos, while 10,170 cycles (73.11%) contained at least one good-quality embryo. The overall clinical pregnancy and live birth rates were 51.09% and 41.04%, respectively. The miscarriage rate among clinical pregnancies was 16.30%, with early and late miscarriage rates of 11.71% and 4.59% (Table 1).

**Table 1:** Patients characteristics and pregnancy outcomes

Characteristics	mean ± SD / *N* (%)
No. of patients	13,911
Female age (years)	30.68 ± 4.38
Male age (years)	31.47 ± 4.84
Female BMI (kg/m^2^)	23.31 ± 3.55
Infertility duration (years)	4.34 ± 2.97
Infertility type	
Primary infertility	7394 (53.15%)
Secondary infertility	6517 (46.85%)
Infertility cause	
Tubal factor	7139 (51.32%)
PCOS	1256 (9.03%)
Endometriosis	597 (4.29%)
Diminished ovarian reserve	361 (2.60%)
Male factor	2001 (14.38%)
Combination	1559 (11.21%)
Unexplained	998 (7.17%)
Total AFC	13.50 ± 7.49
Gravidity	
0	7394 (53.15%)
1	3565 (25.63%)
≥ 2	2952 (21.22%)
Parity	
0	11,147 (80.13%)
≥ 1	2764 (19.87%)
No. of cycles	
1	9045 (65.02%)
2	3340 (24.01%)
3	1526 (10.97%)
FET protocol	
HRT	10,385 (74.65%)
Down-regulation + HRT	1832 (13.17%)
Natural cycle	1400 (10.06%)
Induced ovulation cycle	294 (2.11%)
Endometrium thickness (mm)	9.63 ± 1.65
No. of embryo transfer	
1	2872 (20.65%)
2	11,039 (79.35%)
Stage of embryo	
Cleavage stage	10,948 (78.70%)
Blastocyst	2963 (21.30%)
Grade of embryo	
no good embryo	3741 (26.89%)
≥ 1 good embryo	10,170 (73.11%)
Biochemical pregnancy	7550 (54.27%)
Clinical pregnancy	7107 (51.09%)
Type of clinical pregnancy	
Intrauterine pregnancy	6926 (49.79%)
Ectopic pregnancy	155 (1.11%)
Ectopic and intrauterine pregnancy	26 (0.19%)
No of sac	
1	4657 (33.48%)
2	2295 (16.50%)
No. of fetal heart	
1	4508 (32.41%)
2	2000 (14.38%)
Implantation	9247 (37.06%)
Early miscarriage	832 (11.71%)
Late miscarriage	326 (4.59%)
Live birth	5709 (41.04%)

*BMI* body mass index, *AFC* antral follicle count, *PCOS* polycystic ovary syndrome, *HRT* hormone replacement treatment

### The effect of BMI on pregnancy outcomes

In crude analysis, pregnancy outcomes differed significantly across BMI categories. Women with higher BMI exhibited higher biochemical pregnancy rates (*P* = 0.004), clinical pregnancy rates (*P* = 0.007), and implantation rates (*P* < 0.001). However, early miscarriage rates increased markedly from 8.96% to 17.49% (*P* = 0.001), and late miscarriage rates from 1.65% to 9.04% (*P* < 0.001) across BMI categories (Table 2).

**Table 2:** Pregnancy outcomes, stratified by female BMI (kg/m^2^)

Pregnancy outcomes	BMI < 18.5	18.5 ≤ BMI < 25	25 ≤ BMI < 30	BMI ≥ 30	*P*-value
N	875	8925	3492	619	
Female age (years)	29.68 ± 3.85	30.70 ± 4.39	30.96 ± 4.49	30.39 ± 4.18	< 0.001
Biochemical pregnancy (%)	448 (51.20%)	4783 (53.59%)	1959 (56.10%)	360 (58.16%)	0.004
Clinical pregnancy (%)	424 (48.46%)	4501 (50.43%)	1839 (52.66%)	343 (55.41%)	0.007
Implantation (%)	537 (34.29%)	5856 (36.57%)	2412 (38.52%)	442 (39.89%)	< 0.001
Early miscarriage (%)	38 (8.96%)	508 (11.29%)	226 (12.29%)	60 (17.49%)	0.001
Late miscarriage (%)	7 (1.65%)	167 (3.71%)	121 (6.58%)	31 (9.04%)	< 0.001
Live birth (%)	366 (41.83%)	3660 (41.01%)	1441 (41.27%)	242 (39.10%)	0.736

Early or late miscarriage rate are calculated among clinical pregnancies. Distributions were compared using One-way ANOVA. Categorical variables were compared using Pearson’s chi-square test

Data are presented as either means ± SD or number (%)

After multivariable adjustment using GEE models, obese women (BMI ≥ 30 kg/m²) demonstrated significantly increased risks of early miscarriage (adjusted odds ratios (aOR) 1.74, 95% CI 1.31–2.31), late miscarriage (aOR 2.35, 95% CI 1.55–3.57), and total miscarriage (aOR 2.04, 95% CI 1.58–2.62) compared with normal-weight women. Overweight women (BMI 25.0–29.9 kg/m²) exhibited elevated risks of late miscarriage (aOR 1.66, 95% CI 1.30–2.12) and total miscarriage (aOR 1.30, 95% CI 1.13–1.50), but not early miscarriage (aOR 1.12, 95% CI 0.95–1.33). No statistically significant associations were observed between BMI and clinical pregnancy or live birth after adjustment. Underweight showed no significant association with any pregnancy outcome (Table 3).

**Table 3:** Multivariate generalized estimating equation (GEE) analysis of female BMI to pregnancy outcomes

Variable	Non-adjusted		Adjust	
	Crude OR (95% CI)	*P*-value	Adjusted OR (95% CI)	*P*-value
Biochemical pregnancy				
BMI < 18.5 (kg/m^2^)	0.91 (0.79, 1.04)	0.176	0.88 (0.76, 1.02)	0.098
18.5 ≤ BMI < 25 (kg/m^2^)	1		1	
25 ≤ BMI < 30 (kg/m^2^)	1.11 (1.02, 1.20)	0.012	1.10 (1.01, 1.19)	**0.032**
BMI ≥ 30 (kg/m^2^)	1.20 (1.02, 1.42)	0.028	1.15 (0.97, 1.37)	0.106
Clinical pregnancy				
BMI < 18.5 (kg/m^2^)	0.92 (0.80, 1.06)	0.265	0.89 (0.77, 1.03)	0.131
18.5 ≤ BMI < 25 (kg/m^2^)	1		1	
25 ≤ BMI < 30 (kg/m^2^)	1.09 (1.01, 1.18)	0.025	1.08 (1.00, 1.18)	0.053
BMI ≥ 30 (kg/m^2^)	1.22 (1.04, 1.44)	0.017	1.18 (0.99, 1.40)	0.067
Early miscarriage				
BMI < 18.5 (kg/m^2^)	0.77 (0.55, 1.09)	0.154	0.80 (0.56, 1.14)	0.218
18.5 ≤ BMI < 25 (kg/m^2^)	1		1	
25 ≤ BMI < 30 (kg/m^2^)	1.10 (0.93, 1.30)	0.258	1.12 (0.95, 1.33)	0.178
BMI ≥ 30 (kg/m^2^)	1.67 (1.24, 2.23)	< 0.001	1.74 (1.31, 2.31)	**< 0.001**
Late miscarriage				
BMI < 18.5 (kg/m^2^)	0.44 (0.20, 0.93)	0.033	0.46 (0.21, 1.00)	0.051
18.5 ≤ BMI < 25 (kg/m^2^)	1		1	
25 ≤ BMI < 30 (kg/m^2^)	1.83 (1.44, 2.32)	< 0.001	1.66 (1.30, 2.12)	**< 0.001**
BMI ≥ 30 (kg/m^2^)	2.58 (1.73, 3.85)	< 0.001	2.35 (1.55, 3.57)	**< 0.001**
Total miscarriage				
BMI < 18.5 (kg/m^2^)	0.71 (0.51, 0.98)	0.037	0.73 (0.53, 1.01)	0.056
18.5 ≤ BMI < 25 (kg/m^2^)	1		1	
25 ≤ BMI < 30 (kg/m^2^)	1.32 (1.14, 1.52)	< 0.001	1.30 (1.13, 1.50)	**< 0.001**
BMI ≥ 30 (kg/m^2^)	2.05 (1.59, 2.64)	< 0.001	2.04 (1.58, 2.62)	**< 0.001**
Live birth				
BMI < 18.5 (kg/m^2^)	1.03 (0.90, 1.19)	0.638	1.00 (0.86, 1.15)	0.964
18.5 ≤ BMI < 25 (kg/m^2^)	1		1	
25 ≤ BMI < 30 (kg/m^2^)	1.01 (0.93, 1.09)	0.793	1.01 (0.93, 1.10)	0.771
BMI ≥ 30 (kg/m^2^)	0.92 (0.78, 1.09)	0.349	0.90 (0.76, 1.07)	0.222

Data were analyzed using GEE models to account for multiple cycles from the same woman. Bold indicates *P* < 0.05 after multivariable adjustment

For early/ late/ total miscarriage, analyses were performed among clinical pregnancies. Model adjusted for: female age; infertility type; infertility duration; infertility cause; FET protocol; endometrium thickness; number of embryo transfer; embryo stage; embryo grade

*BMI* body mass index

### Effect modification by pregnancy plurality

In women with elevated BMI, twin gestation is associated with a higher risk of total miscarriage compared with singleton pregnancy. For total miscarriage, the aOR for overweight and obese women were 1.21 (95% CI 0.99–1.48) and 2.25 (95% CI 1.59–3.19) among singleton pregnancies, respectively, while the corresponding values were 1.96 (95% CI 1.36–2.83) and 2.50 (95% CI 1.29–4.84) in twin pregnancies (*P* for interaction = 0.018).

For early and late miscarriage, no significant interactions were observed between BMI and plurality. The aOR for early miscarriage were 1.04 (95% CI 0.81–1.34) and 1.99 (95% CI 1.33–2.99) in singleton pregnancies for overweight and obese women, respectively, compared with 1.61 (95% CI 0.80–3.24) and 1.65 (95% CI 0.38–7.04) in twin pregnancies (*P* for interaction = 0.198). For late miscarriage, the aORs were 1.43 (95% CI 1.06–1.95) and 2.30 (95% CI 1.38–3.82) for overweight and obese women in singleton pregnancies, while the corresponding values were 1.96 (95% CI 1.28–2.99) and 2.61 (95% CI 1.23–5.52) in twin pregnancies (*P* for interaction = 0.314) (Table 4). Crude miscarriage rates for singleton and twin pregnancies stratified by BMI are shown in Supplementary Table 1.

**Table 4:** Association of BMI with early/ late/ total miscarriage rate stratified by No. of fetal heartbeats

Variable	BMI, kg/m^2^				*P* for interaction
	< 18.5	18.5 to < 25	25 to < 30	≥ 30	
Early miscarriage					0.198
Singleton pregnancy	0.83 (0.50, 1.37)	1	1.04 (0.81, 1.34)	1.99 (1.33, 2.99)	
Twin pregnancy	3.08 (1.04, 9.07)	1	1.61 (0.80, 3.24)	1.65 (0.38, 7.04)	
Late miscarriage					0.314
Singleton pregnancy	0.42 (0.17, 1.04)	1	1.43 (1.06, 1.95)	2.30 (1.38, 3.82)	
Twin pregnancy	0.45 (0.11, 1.83)	1	1.96 (1.28, 2.99)	2.61 (1.23, 5.52)	
Total miscarriage					**0.018**
Singleton pregnancy	0.68 (0.44, 1.05)	1	1.21 (0.99, 1.48)	2.25 (1.59, 3.19)	
Twin pregnancy	1.07 (0.46, 2.52)	1	1.96 (1.36, 2.83)	2.50 (1.29, 4.84)	

Analyses were performed among pregnancies with identifiable fetal heartbeat(s). Odds ratios were derived from generalized estimating equation (GEE) models adjusted for the same covariates as in Table 3. The *P* value for interaction was obtained by including an interaction term between BMI categories and plurality in the GEE model. Bold indicates *P* < 0.05

Sensitivity analyses were carried out to test the robustness of the main results, using Asian BMI classification criteria and excluding cases with spontaneous twin reduction at 4–5 weeks after embryo transfer. Statistically significant interactions were observed between BMI and singleton/twin pregnancy regarding total miscarriage risk (*P* for interaction = 0.007 and 0.023, respectively), and these findings remained consistent across the analyses (Supplementary Table 2 and Supplementary Table 3).

## Discussion

In this large retrospective cohort study of 13,911 FET cycles, we found that elevated maternal BMI was significantly associated with an increased risk of miscarriage. Notably, this association was stronger in twin pregnancies than in singleton pregnancies, suggesting that pregnancy plurality may modify the relationship between BMI on miscarriage risk. Despite a trend toward higher clinical pregnancy rates in women with elevated BMI in unadjusted analyses, this rate was comparable across BMI groups after multivariable adjustment. These findings provide additional evidence supporting the detrimental influence of maternal obesity on miscarriage and suggest that the physiological burden of twin gestation may contribute to obesity-related miscarriage risk.

Our findings demonstrated that obese women had a 74% increased risk of early miscarriage and more than a twofold increased risk of late miscarriage relative to normal-weight women after confounder adjustment, while overweight women had a 66% increased risk of late miscarriage. These results are consistent with previous studies in assisted reproductive technology populations [2, 16–18]. Underweight status showed no significant association with adverse pregnancy outcomes after adjustment, which was also consistent with previous reports [19]. However, elevated BMI did not significantly reduce the live birth rate. This was probably due to a higher clinical pregnancy rate in overweight (adjusted OR = 1.08, *P* = 0.053) and obese women (adjusted OR = 1.18, *P* = 0.067), which, despite lacking statistical significance, numerically offset the adverse effect of the increased miscarriage rate on live birth.

Twin pregnancy is also an established risk factor for adverse pregnancy outcomes, including pregnancy loss [20]. However, few studies have formally tested whether pregnancy plurality modifies the effect of BMI on miscarriage. Qu et al. reported significant interactions between BMI and pregnancy plurality for miscarriage in an ART population, but their study had several limitations, including no separate assessment of late miscarriage, mixing of fresh and frozen-thawed cycles, and pooling overweight with obesity into one category [21]. Our study addresses these gaps by focusing exclusively on FET cycles, distinguishing early from late miscarriage, and analyzing overweight as an independent category. Notably, we found that overweight women did not show a significantly elevated risk of total miscarriage in singleton pregnancy but had a substantially higher risk in twin pregnancy, whereas obese women exhibited such an elevated risk in both pluralities.

When stratified by pregnancy plurality, we found that the incremental risk of miscarriage attributable to BMI was significantly larger in twin gestations relative to singleton pregnancies. This suggests that twin gestation may lower the physiological threshold at which overweight becomes a detrimental factor for pregnancy maintenance. However, the interaction between BMI and pregnancy plurality was statistically significant only for total miscarriage (*P* for interaction = 0.018), but not for early or late miscarriage. This may be due to limited statistical power in the subgroup analyses. Total miscarriage may therefore provide a more sensitive measure of this interaction. Nevertheless, obese women had an elevated risk of miscarriage in both singleton and twin pregnancies, and the overlapping confidence intervals and wide subgroup confidence intervals warrant cautious interpretation. Larger studies are needed to validate these findings.

Mechanistically, elevated BMI reflects metabolic disturbances (e.g., insulin resistance, chronic inflammation, adipokine dysregulation) that impair endometrial receptivity, trophoblast invasion, and placentation [22–24]. Such early placental compromise predisposes to obesity-related complications (e.g., hypertensive disorders) and late miscarriage [25, 26]. Twin pregnancies impose greater metabolic and hemodynamic demands than singletons, including larger placental mass and more pronounced insulin resistance [27–31]. When obesity-related disturbances coexist with twin gestation, these physiological alterations may synergistically worsen placental insufficiency and increase pregnancy loss [25, 32].

Our findings have several clinical implications for patients undergoing FET. First, in overweight and obese patients, the results support consideration of eSET as a strategy to potentially reduce the miscarriage risk associated with elevated BMI. This approach may also lower the risks of preterm birth, placental complications, and other obesity-related complications (e.g., gestational diabetes, and macrosomia) [33, 34]. Second, preconception weight optimization is critically important. Achieving a normal BMI should be strongly recommended to improve metabolic health and pregnancy maintenance, especially if double embryo transfer is planned. These findings provide novel evidence in support of BMI-based embryo transfer strategies and associated clinical management.

The major strengths of this study are as follows. First, the large sample size provided sufficient statistical power to detect interaction effects. Second, the distinction between early and late miscarriage allowed for more refined risk assessment. Third, the use of GEE models accounted for repeated cycles from the same patient, enabling more robust estimates. To our knowledge, this is the first large cohort study to formally examine how maternal BMI and pregnancy plurality interact to affect miscarriage risk in FET. Several limitations should also be noted. The retrospective, single-center design and lack of longitudinal BMI data may limit generalizability and introduce residual confounding. Smoking data were unavailable. Although active smoking is rare among Chinese women, passive exposure and male smoking remain potential confounders. The sample sizes were relatively small in some subgroup, especially in twin pregnancies for obese women, which reduces the precision of effect estimates. And stratification by plurality may introduce collider bias as plurality is determined post-implantation and influenced by BMI. Thus, the interaction should be interpreted with caution. Finally, the findings are specific to FET cycles, where placental adaptation may differ from fresh transfers as evidenced by differential fetal crown-rump length growth [35]. Accordingly, extrapolation to fresh cycles warrants caution.

Future research directions include prospective studies to validate our findings, mechanistic research on the placental-metabolic interface in obese women with twin pregnancies, and preconception weight loss interventions. Multicenter studies with larger numbers of obese women and twin pregnancies are needed to confirm the robustness of the interaction and to further evaluate the role of eSET in this population.

In conclusion, elevated maternal BMI is associated with increased miscarriage risk following FET, with this association may significantly modified by pregnancy plurality and being stronger in twin pregnancies. These findings support preconception weight management and eSET in overweight and obese patients.

## Supplementary Information

Supplementary Material 1.

Supplementary Material 2.

## Data Availability

The data are available upon reasonable request from the corresponding author.

## Abbreviations

ART: Assisted reproductive technologies
FET: Frozen embryo transfer
BMI: Body mass index
AFC: Antral follicle count
PCOS: Polycystic ovary syndrome
eSET: Elective single embryo transfer
PGT: Preimplantation genetic testing
GnRH: Gonadotropin-releasing hormone
HRT: Hormone replacement therapy
GEE: Generalized estimating equation
OR: Odds ratios
CI: Confidence intervals
aOR: Adjusted odds ratios

## References

[1] Wei D, Liu JY, Sun Y, Shi Y, Zhang B, Liu JQ, et al. Frozen versus fresh single blastocyst transfer in ovulatory women: a multicentre, randomised controlled trial. Lancet. 2019;393(10178):1310–8. 10.1016/S0140-6736(18)32843-5 30827784

[2] Fabozzi G, Cimadomo D, Maggiulli R, Vaiarelli A, Badajoz V, Aura M, et al. Association between oocyte donors’ or recipients’ body mass index and clinical outcomes after first single blastocyst transfers-the uterus is the most affected. Fertil Steril. 2024;121(2):281–90. 10.1016/j.fertnstert.2023.07.029 37549838

[3] Boots C, Stephenson MD. Does obesity increase the risk of miscarriage in spontaneous conception: a systematic review. Semin Reprod Med. 2011;29(6):507–13. 10.1055/s-0031-1293204 22161463

[4] Lee R, Brandt JS, Ananth CV. Placental abruption and perinatal mortality in twins: novel insight into management at preterm versus term gestations. Eur J Epidemiol. 2024;39(11):1267–76. 10.1007/s10654-024-01171-z PMC11646271 39576360

[5] Luke B, Brown MB, Stern JE, Missmer SA, Fujimoto VY, Leach R, et al. Female obesity adversely affects assisted reproductive technology (ART) pregnancy and live birth rates. Hum Reprod. 2011;26(1):245–52. 10.1093/humrep/deq306 21071489

[6] Larsen NL, Koefoed AS, Kampmann U, Retnakaran R, Ovesen PG, Fuglsang J. Gestational diabetes mellitus in twin pregnancies versus singleton pregnancies-A retrospective case-control study. Diabet Med. 2025;42(10):e70095. 10.1111/dme.70095 PMC12434430 40548386

[7] Practice Committee of the American Society for Reproductive Medicine. Recurrent pregnancy loss: a committee opinion. Fertil Steril. 2026;125(6):1023–41. 10.1016/j.fertnstert.2026.03.001 42062119

[8] Barbhaiya M, Zuily S, Naden R, Hendry A, Manneville F, Amigo MC, et al. The 2023 ACR/EULAR Antiphospholipid Syndrome Classification Criteria. Arthritis Rheumatol. 2023;75(10):1687–702. 10.1002/art.42624 37635643

[9] World Health Organization. Obesity: preventing and managing the global epidemic: report of a WHO consultation. WHO Tech Rep Ser. 2000;894:i–xii. 11234459

[10] Consultation WHOE. Appropriate body-mass index for Asian populations and its implications for policy and intervention strategies. Lancet. 2004;363(9403):157–63. 10.1016/S0140-6736(03)15268-3 14726171

[11] Yang AM, Xu X, Han Y, Wei JJ, Hao GM, Cui N, et al. Risk Factors for Different Types of Pregnancy Losses: Analysis of 15,210 Pregnancies After Embryo Transfer. Front Endocrinol (Lausanne). 2021;12:683236. 10.3389/fendo.2021.683236 PMC8267909 34248846

[12] Alpha Scientists in Reproductive M, Embryology ESIGo. The Istanbul consensus workshop on embryo assessment: proceedings of an expert meeting. Hum Reprod. 2011;26(6):1270–83. 10.1093/humrep/der037 21502182

[13] Gardner DK, Lane M, Stevens J, Schlenker T, Schoolcraft WB. Blastocyst score affects implantation and pregnancy outcome: towards a single blastocyst transfer. Fertil Steril. 2000;73(6):1155–8. 10.1016/s0015-0282(00)00518-5 10856474

[14] Working Group on the update of the ESHRE/ALPHA Istanbul Consensus, Coticchio G, Ahlström A, Arroyo G, Balaban B, Campbell A, et al. The Istanbul Consensus update: a revised ESHRE/ALPHA consensus on oocyte and embryo static and dynamic morphological assessment† ‡. Reprod Biomed Online. 2025;50(6):104955. 10.1016/j.rbmo.2025.104955 40300986

[15] Talbot AL, Alexopoulou E, Kallemose T, Freiesleben NC, Nielsen HS, Zedeler A. Binucleated embryos at the two-cell stage show higher blastocyst formation rates and higher pregnancy and live birth rates compared to non-multinucleated embryos. Hum Reprod Open. 2022;2022(4):hoac049. 10.1093/hropen/hoac049 PMC9700381 36452346

[16] Sarais V, Pagliardini L, Rebonato G, Papaleo E, Candiani M, Vigano P. A Comprehensive Analysis of Body Mass Index Effect on in Vitro Fertilization Outcomes. Nutrients. 2016;8(3):109. 10.3390/nu8030109 PMC4808839 26907340

[17] Bellver J. BMI and miscarriage after IVF. Curr Opin Obstet Gynecol. 2022;34(3):114–21. 10.1097/GCO.0000000000000778 35645009

[18] Song Z, Fang F, Liu Z, Liang R, Pan Y, Meng W, et al. Elevated triglyceride glucose-body mass index increases risk of miscarriage in women undergoing in vitro fertilization and embryo transfer. Fertil Steril. 2026;125(3):466–76. 10.1016/j.fertnstert.2025.09.001 40935277

[19] Romanski PA, Bortoletto P, Chung A, Magaoay B, Rosenwaks Z, Spandorfer SD. Reproductive and obstetric outcomes in mildly and significantly underweight women undergoing IVF. Reprod Biomed Online. 2021;42(2):366–74. 10.1016/j.rbmo.2020.10.011 33243662

[20] Whittaker M, Greatholder I, Kilby MD, Heazell AEP. Risk factors for adverse outcomes in twin pregnancies: a narrative review. J Matern Fetal Neonatal Med. 2023;36(2):2240467. 10.1080/14767058.2023.2240467 37518183

[21] Qu P, Yan M, Zhao D, Wang D, Dang S, Shi W, et al. Association Between Pre-Pregnancy Body Mass Index and Miscarriage in an Assisted Reproductive Technology Population: A 10-Year Cohort Study. Front Endocrinol (Lausanne). 2021;12:646162. 10.3389/fendo.2021.646162 PMC8242335 34220704

[22] Lei R, Chen S, Li W. Advances in the study of the correlation between insulin resistance and infertility. Front Endocrinol (Lausanne). 2024;15:1288326. 10.3389/fendo.2024.1288326 PMC10860338 38348417

[23] Galio L, Bernet L, Rodriguez Y, Fourcault C, Dieudonne MN, Pinatel H, et al. The effect of obesity on uterine receptivity is mediated by endometrial extracellular vesicles that control human endometrial stromal cell decidualization and trophoblast invasion. J Extracell Biol. 2023;2(7):e103. 10.1002/jex2.103 PMC11080792 38939074

[24] Wang Y, Chen Z, Wang R, Song A, Zhang Y. Obesity and oxidative stress: potential mechanisms in endometrial disorders. Front Endocrinol (Lausanne). 2026;17:1709556. 10.3389/fendo.2026.1709556 PMC12932235 41757245

[25] Leung TY, Leung TN, Sahota DS, Chan OK, Chan LW, Fung TY, et al. Trends in maternal obesity and associated risks of adverse pregnancy outcomes in a population of Chinese women. BJOG. 2008;115(12):1529–37. 10.1111/j.1471-0528.2008.01931.x 19035989

[26] Langley-Evans SC, Pearce J, Ellis S. Overweight, obesity and excessive weight gain in pregnancy as risk factors for adverse pregnancy outcomes: A narrative review. J Hum Nutr Diet. 2022;35(2):250–64. 10.1111/jhn.12999 PMC9311414 35239212

[27] McFadyen M, Farquharson J, Cockburn F. Maternal and umbilical cord erythrocyte omega-3 and omega-6 fatty acids and haemorheology in singleton and twin pregnancies. Arch Dis Child Fetal Neonatal Ed. 2003;88(2):F134–8. 10.1136/fn.88.2.F134 PMC1721517 12598503

[28] Ouyang Y, Cai P, Gong F, Lin G, Qin J, Li X. The risk of twin pregnancies should be minimized in patients with a unicornuate uterus undergoing IVF-ET. Sci Rep. 2020;10(1):5571. 10.1038/s41598-020-62311-5 PMC7101414 32221343

[29] Mourad M, Too G, Gyamfi-Bannerman C, Zork N. Hypertensive disorders of pregnancy in twin gestations complicated by gestational diabetes(). J Matern Fetal Neonatal Med. 2021;34(5):720–4. 10.1080/14767058.2019.1614160 31096815

[30] Tang WZ, Cai QY, Wang YH, Chen Y, Lan X, Wang YX, et al. Risk of neonatal adverse pregnancy outcome in twin pregnancies conceived via in-vitro fertilization. Reprod Health. 2025. https://link.springer.com/article/10.1186/s12978-025-02139-2. Epub ahead of print. 10.1186/s12978-025-02139-2 41449465

[31] Santana DS, Surita FG, Cecatti JG. Multiple Pregnancy: Epidemiology and Association with Maternal and Perinatal Morbidity. Rev Bras Ginecol Obstet. 2018;40(9):554–62. 10.1055/s-0038-1668117 PMC10316907 30231294

[32] Cao Y, Ding M, Chen J, Zhang C, He F, Li X, et al. The relationship between insulin resistance and recurrent pregnancy loss in assisted reproductive technology: A retrospective case-control study. Med (Baltim). 2025;104(22):e42373. 10.1097/MD.0000000000042373 PMC12129513 40441206

[33] Kamath MS, Mascarenhas M, Kirubakaran R, Bhattacharya S. Number of embryos for transfer following in vitro fertilisation or intra-cytoplasmic sperm injection. Cochrane Database Syst Rev. 2020;8(8):CD003416. 10.1002/14651858.CD003416.pub5 PMC8094586 32827168

[34] Pinborg A. IVF/ICSI twin pregnancies: risks and prevention. Hum Reprod Update. 2005;11(6):575–93. 10.1093/humupd/dmi027 16123055

[35] Cavoretto P, Farina A, Girardelli S, et al. Greater fetal crown-rump length growth with the use of in vitro fertilization or intracytoplasmic sperm injection conceptions after thawed versus fresh blastocyst transfers: secondary analysis of a prospective cohort study. Fertil Steril. 2021;116:147–56. 10.1016/j.fertnstert.2020.11.035 33500139

