# Supplementary material for: Modification effect of multiple gestation on the relationship between maternal body mass index and miscarriage in frozen-thawed embryo transfer: a retrospective cohort study
Source: Reprod Biol Endocrinol. 2026 Jul 4;24:89. doi: 10.1186/s12958-026-01586-1 (PMC13613619; doi:10.1186/s12958-026-01586-1)
Supplement: Supplementary file 1 [file 12958_2026_1586_MOESM2_ESM.docx]

Supplementary Table 1 Crude early/ late/ total miscarriage rate stratified by BMI and number of fetal heartbeats.

| Variable | BMI, kg/m^2^ | | | | *P* |
| --- | --- | --- | --- | --- | --- |
|  | <18.5 | 18.5 to <25 | 25 to <30 | ≥30 |  |
| **Early miscarriage** |  |  |  |  |  |
| Singleton pregnancy | 18 (6.19%) | 213 (7.53%) | 90 (7.71%) | 29 (13.00%) | 0.021 |
| Twin pregnancy | 4 (3.77%) | 20 (1.55%) | 14 (2.70%) | 2 (2.30%) | 0.224 |
| **Late miscarriage** |  |  |  |  |  |
| Singleton pregnancy | 5 (1.72%) | 113 (4.00%) | 70 (6.00%) | 20 (8.97%) | <0.001 |
| Twin pregnancy | 2 (1.89%) | 54 (4.19%) | 50 (9.65%) | 11 (12.64%) | <0.001 |
| **Total miscarriage** |  |  |  |  |  |
| Singleton pregnancy | 23 (7.90%) | 326 (11.53%) | 160 (13.71%) | 49 (21.97%) | <0.001 |
| Twin pregnancy | 6 (5.66%) | 74 (5.74%) | 64 (12.36%) | 13 (14.94%) | <0.001 |

Data are presented as number (%).

Percentages are calculated among clinical pregnancies in each subgroup (singleton or twin pregnancies). Categorical variables were compared using Pearson’s chi-square test.

Supplementary Table 2 Association of BMI (Asian-specific criteria) with early/ late/ total miscarriage rate stratified by No. of fetal heartbeats.

| Variable | BMI, kg/m^2^ | | | | *P* for interaction |
| --- | --- | --- | --- | --- | --- |
|  | <18.5 | 18.5 to <23 | 23 to <27.5 | ≥27.5 |  |
| **Early miscarriage** |  |  |  |  | 0.068 |
| Singleton pregnancy | 0.81 (0.49, 1.34) | 1 | 0.90 (0.70, 1.16) | 1.53 (1.12, 2.09) |  |
| Twin pregnancy | 3.77 (1.19, 11.93) | 1 | 1.87 (0.91, 3.84) | 1.91 (0.68, 5.37) |  |
| **Late miscarriage** |  |  |  |  | 0.080 |
| Singleton pregnancy | 0.42 (0.17, 1.04) | 1 | 1.18 (0.86, 1.61) | 1.69 (1.14, 2.51) |  |
| Twin pregnancy | 0.47 (0.11, 1.98) | 1 | 1.48 (0.93, 2.35) | 3.16 (1.86, 5.36) |  |
| **Total miscarriage** |  |  |  |  | **0.007** |
| Singleton pregnancy | 0.66 (0.42, 1.03) | 1 | 0.99 (0.81, 1.22) | 1.66 (1.28, 2.15) |  |
| Twin pregnancy | 1.21 (0.50, 2.91) | 1 | 1.60 (1.08, 2.38) | 3.01 (1.88, 4.82) |  |

Analyses were performed among pregnancies with identifiable fetal heartbeat(s). Odds ratios were derived from generalized estimating equation (GEE) models adjusted for the same covariates as in Table 3. The *P* value for interaction was obtained by including an interaction term between BMI categories and plurality in the GEE model.

Supplementary Table 3 Association of BMI with early/ late/ total miscarriage rate stratified by No. of fetal heartbeats (excluding spontaneous twin reduction at 4-5 weeks after transfer).

| Variable | BMI, kg/m^2^ | | | | *P* for interaction |
| --- | --- | --- | --- | --- | --- |
|  | <18.5 | 18.5 to <25 | 25 to <30 | ≥30 |  |
| **Early miscarriage** |  |  |  |  | 0.214 |
| Singleton pregnancy | 0.85 (0.51, 1.42) | 1 | 1.08 (0.83, 1.40) | 2.14 (1.41, 3.26) |  |
| Twin pregnancy | 3.08 (1.04, 9.07) | 1 | 1.61 (0.80, 3.24) | 1.65 (0.38, 7.04) |  |
| **Late miscarriage** |  |  |  |  | 0.319 |
| Singleton pregnancy | 0.35 (0.13, 0.96) | 1 | 1.45 (1.05, 1.99) | 2.39 (1.42, 4.02) |  |
| Twin pregnancy | 0.45 (0.11, 1.83) | 1 | 1.96 (1.28, 2.99) | 2.61 (1.23, 5.52) |  |
| **Total miscarriage** |  |  |  |  | **0.023** |
| Singleton pregnancy | 0.66 (0.42, 1.05) | 1 | 1.24 (1.01, 1.53) | 2.41 (1.69, 3.45) |  |
| Twin pregnancy | 1.07 (0.46, 2.52) | 1 | 1.96 (1.36, 2.83) | 2.50 (1.29, 4.84) |  |

Analyses were performed among pregnancies with identifiable fetal heartbeat(s), excluding cases of spontaneous twin reduction at 4-5 weeks after transfer. Odds ratios were derived from generalized estimating equation (GEE) models adjusted for the same covariates as in Table 3. The *P* value for interaction was obtained by including an interaction term between BMI categories and plurality in the GEE model.
